# Rapid Birth–Death Evolution Specific to Xenobiotic Cytochrome P450 Genes in Vertebrates

**DOI:** 10.1371/journal.pgen.0030067

**Published:** 2007-05-11

**Authors:** James H Thomas

**Affiliations:** Department of Genome Sciences, University of Washington, Seattle, Washington, United States of America; Fred Hutchinson Cancer Research Center, United States of America

## Abstract

Genes vary greatly in their long-term phylogenetic stability and there exists no general explanation for these differences. The cytochrome P450 *(CYP450)* gene superfamily is well suited to investigating this problem because it is large and well studied, and it includes both stable and unstable genes. *CYP450* genes encode oxidase enzymes that function in metabolism of endogenous small molecules and in detoxification of xenobiotic compounds. Both types of enzymes have been intensively studied. My analysis of ten nearly complete vertebrate genomes indicates that each genome contains 50–80 *CYP450* genes, which are about evenly divided between phylogenetically stable and unstable genes. The stable genes are characterized by few or no gene duplications or losses in species ranging from bony fish to mammals, whereas unstable genes are characterized by frequent gene duplications and losses (birth–death evolution) even among closely related species. All of the *CYP450* genes that encode enzymes with known endogenous substrates are phylogenetically stable. In contrast, most of the unstable genes encode enzymes that function as xenobiotic detoxifiers. Nearly all unstable *CYP450* genes in the mouse and human genomes reside in a few dense gene clusters, forming unstable gene islands that arose by recurrent local gene duplication. Evidence for positive selection in amino acid sequence is restricted to these unstable *CYP450* genes, and sites of selection are associated with substrate-binding regions in the protein structure. These results can be explained by a general model in which phylogenetically stable genes have core functions in development and physiology, whereas unstable genes have accessory functions associated with unstable environmental interactions such as toxin and pathogen exposure. Unstable gene islands in vertebrates share some functional properties with bacterial genomic islands, though they arise by local gene duplication rather than horizontal gene transfer.

## Author Summary

Genes vary greatly in their long-term phylogenetic stability, and there exists no general explanation for these differences. Stable genes persist as a single copy over a wide range of distantly related species, whereas unstable genes undergo frequent duplication and loss in a process called birth-death evolution. The vertebrate cytochrome P450 *(CYP450)* gene superfamily includes many genes that are present in a single copy in species ranging from teleost fish to mammals and other groups of genes that undergo active birth-death evolution across the same species. The author found that nearly all stable *CYP450* genes encode enzymes known to function in the synthesis and degradation of steroid and retinoid hormones (and related molecules). These hormones function in core developmental pathways in vertebrates. In contrast, most unstable *CYP450* genes encode enzymes that detoxify foreign small molecules (called xenobiotics—foreign biochemicals). In addition, many of the unstable *CYP450* genes are subject to natural selection to change their amino acid sequence over time (positive selection), probably in response to changes in xenobiotic exposure. These findings suggest that stable and unstable genes differ in their rates of birth-death evolution, because stable genes have core endogenous functions whereas unstable genes respond to changing environmental conditions.

## Introduction

Genes in animal and plant gene families vary greatly in their phylogenetic stability. Some genes persist as a single copy over a wide phylogenetic range of species, with few or no gene duplications or losses on different evolving lineages. Other genes undergo frequent duplication and loss in a process called birth-death evolution [1,2]. Gene duplication and subsequent divergence of the duplicate copies underlie the formation of gene families and are thought to be an important source of genetic complexity and evolutionary change [3–5]. Though many specific examples of stable and unstable genes have been documented, there exists no general explanation for their different patterns of evolution. To explore the evolutionary basis for these patterns, I sought a gene family with characteristics that make it amenable to detailed analysis and interpretation. The vertebrate cytochrome P450 *(CYP450)* gene superfamily is well suited for the following reasons. First, it is a large family with both phylogenetically stable members and unstable members. Second, the biochemical and organismal functions of most genes in the family have been extensively studied in humans and rodents. Third, their protein products are large and have a well-conserved domain content, providing abundant information for studies of molecular evolution. Finally, strong interest in the family has resulted in relatively high quality annotations of gene structure in a wide range of vertebrate species.

The human genome contains approximately 60 *CYP450* genes, which encode membrane-bound oxidase enzymes that act on a wide variety of substrates (recent reviews include [6–8]). The CYP450 enzymes can be divided into those that act on endogenous small molecules and those that act on xenobiotic compounds. The endogenous-substrate enzymes function in biosynthesis or catabolism of steroids, sterols, retinoids, prostaglandins, and fatty acids. The xenobiotic-substrate enzymes are expressed primarily in the liver and epithelial tissues and function to defend against environmental toxins and carcinogens. These xenobiotic *CYP450* genes have been studied extensively in humans because they comprise one of the major (and most polymorphic) activities that determine drug half-life and are thus important in pharmaceutical development [6,7]. Xenobiotic CYP450 enzymes have also been studied extensively because in some cases they convert exogenous compounds into active carcinogens, presumably as a negative side-effect of broad substrate specificity [9]. Approximately 22 of the human CYP450 enzymes are thought to act primarily on endogenous substrates and approximately 15 other CYP450 enzymes are thought to act primarily on xenobiotic substrates (Figure S1). A few of the remaining enzymes are reported to have activity toward both endogenous and xenobiotic substrates; it is unclear whether one or the other activity (or both) is their primary function. Finally, there are about ten CYP450 enzymes with no known activities, most of which have been identified only recently from human genome sequence.

To study the molecular evolution of the *CYP450* superfamily, I considered for analysis about 20 vertebrate genomes in various stages of draft sequencing, assembly, and annotation. Of these, sequence coverage, assembly, and annotation of 11 genomes were sufficiently complete for my needs. Of these 11, Pan troglodytes was discarded because of its extreme similarity to human, leaving ten nearly complete genomes: six placental mammals, one bird, one amphibian, and two teleost fish. Analysis of gene stability among these genomes reveals that the rate of birth-death evolution among genes acting on xenobiotic substrates is much higher than among those acting on endogenous substrates.

## Results

### Phylogenetic Trees

**Figure 1:**
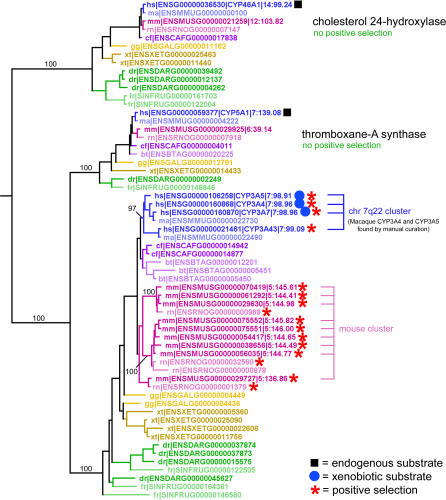
Excerpt from Tree of Vertebrate CYP450 Proteins Shown is one segment of 61 proteins from a maximum-likelihood protein tree of all 628 CYP450 proteins from 10 vertebrate species. The complete tree is shown in Figure S1. Protein names are color coded by species and include a genus-species prefix (e.g., “hs” is H. sapiens). Human and mouse protein names end with the chromosome and position (in Mb) of the corresponding gene. Solid black squares mark human proteins with a known endogenous substrate. Solid blue circles mark human proteins with known xenobiotic substrates. Red asterisks mark genes for which positive selection was detected; tests for positive selection were performed only on primate and rodent genes. Other markings indicate enzyme activity, gene clusters in human (blue), and mouse (pink), and specific groups tested where positive selection was not found (green). Numbers on tree branches show percent bootstrap support for all branches important for interpretation. Almost half of the missing cow *CYP46A1* ortholog is present in the Btau3.1 assembly based on a TBLASTN search (Chromosome 21, plus strand at ~58.4 Mb) (see Table S1). Though not apparent from this tree segment, clear expansions in the number of *CYP450* genes are present on other parts of the complete tree for fish, frog, and chicken (Figure S1).

As starting material, I gathered complete sets of predicted *CYP450* genes from the genomes of human *(Homo sapiens),* rhesus macaque *(Macaca mulatta),* dog *(Canis familiaris),* cow *(Bos taurus),* mouse *(Mus musculus),* rat *(Rattus norvegicus),* chicken *(Gallus gallus)*, clawed frog *(Xenopus tropicalis),* zebrafish *(Danio rerio),* and pufferfish (*Takifugu rubripes)*. All known and predicted splice forms were screened to identify a single canonical coding sequence from each gene for molecular evolutionary analysis (see Materials and Methods). Figure S1 shows a maximum-likelihood protein tree derived from all 628 of these sequences, marked to indicate known or probable functions based on the human proteins. A representative segment of the tree is shown in Figure 1. In the tree, CYP450 enzymes that act on endogenous substrates are generally represented by a single gene in each organism, and the phylogeny of that gene approximates the known species phylogeny. In some cases there are two related proteins in one or both fish species, presumably resulting from the known whole-genome duplication on the fish lineage [10,11]. In all but one case, each orthologous group of genes is strongly separated on the tree from all other *CYP450* genes, indicating that they became functionally specialized well before the divergence of teleost fish from mammals (the single exception is described below). In sharp contrast, nearly all CYP450 enzymes that act on xenobiotic substrates are encoded by genes that have undergone active duplication and loss throughout vertebrate evolution, reflected in multiple species- and lineage-specific gene expansions. The tree segment in Figure 1 includes two endogenous-substrate enzymes and one class of xenobiotic-substrate enzymes; they are typical of the complete tree (Figure S1). Table 1 summarizes gene numbers and variance across species for three groups of *CYP450* genes.

**Table 1:**
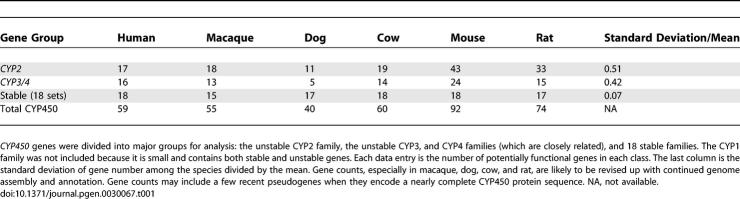
Gene Group Counts

Among the endogenous-substrate ortholog groups, an expected protein from a specific species was occasionally absent, usually from cow, chicken, or frog. One example is found in Figure 1 (a *CYP46A1* cholesterol 24-hydroxylase ortholog is missing from cow). Most of these apparent gene losses probably result from incomplete sequence assemblies or gene annotations. Such apparent gene losses were found most often in the cow, chicken, and frog genomes presumably as a result of relatively immature assemblies and annotations of these genomes. In several cases, translated BLAST nucleotide (TBLASTN) searches of the cognate genome clearly indicated the likely presence of part or all of an unpredicted gene ortholog (see Materials and Methods; Tables S1 and S2). There were also a few cases of apparent species-specific gene duplications outside of fish. For example, there were two predicted rat genes encoding CYP51A1-like proteins (Figure S1). Although these cases may include undiagnosed pseudogenes, it is reasonable to suppose that occasional gene duplications may be tolerated in these groups. Finally, there was one case in which enzymes that act on endogenous substrates (corticosteroid precursors) appear to have become specialized recently and specifically in terrestrial vertebrates (CYP11B1 steroid 11-β-hydroxylase and CYP11B2 aldosterone synthase), probably in connection with resistance to dehydration [12]. Nevertheless, the general picture among endogenous-substrate *CYP450* genes is one of striking phylogenetic stability, with very low rates of gene duplication and loss across jawed vertebrates.

Among CYP450 enzymes with xenobiotic substrates the picture was dramatically different. Almost without exception, one or more species or lineages had multiple genes that grouped in the tree with one or no genes from other species. The sample shown in Figure 1 is typical of the complete tree. These differences in gene content are far too extensive to be accounted for by incomplete genome sequence and annotation. In particular, the human and mouse genomes are especially mature in sequence and annotation, and the pattern of gene duplication and loss is robustly apparent in comparing these two genomes (e.g., Figure 1; Table 2). Extensive bootstrap testing on several subtrees supported the occurrence of independent gene duplications and deletions on both the mouse and human lineages (Figure S2).

**Table 2:**
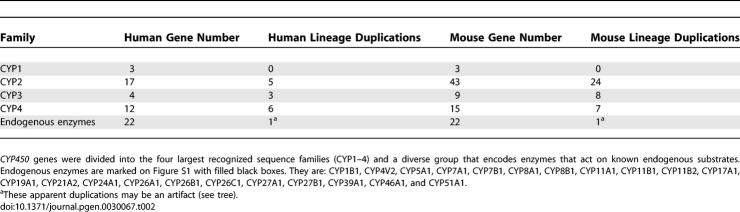
Recent Probable Duplications

In principle, genetic exchange among closely related genes within a species (concerted evolution) could explain some aspects of these patterns by causing lineage-specific clades of genes from sequence homogenization rather than recent duplication. In support of this possibility, a recent paper describes evidence that concerted evolution played a role in the divergence of the *CYP1A1* and *CYP1A2* genes in mammals from their bird orthologs [13]. However there are several reasons to think that this case is unusual. First, all of the syntenic *CYP450* gene clusters between human and mouse contained different numbers of genes, indicating that gene duplication or loss must occur (two examples are shown in Figures S3 and S4). Second, except for a few of the most closely-related gene pairs, nucleotide divergence among duplicate genes was substantial, nucleotide changes were distributed widely across the coding sequence, and regions of higher nucleotide similarity usually coincided with regions of stronger purifying selection in the family in general (unpublished data). Finally, in many cases the tree relationships were inconsistent with concerted evolution being a dominant influence on sequence relatedness (for example, Figure S5). These results suggest that concerted evolution plays, at most, a minor role in shaping evolution in the family as a whole.

### Unstable Genes Are Clustered in the Human and Mouse Genomes

Most human and mouse genes for CYP450 enzymes are scattered widely across their genomes, but there are some exceptions. Clusters of genes are found at human chromosome bands 1p33, 7q22, 10p13, 10q26, and 19q13. In each of these five human clusters, the *CYP450* genes are adjacent, with no other confirmed genes interspersed among them. Within each cluster, all the genes encode closely related proteins with many cases of apparent gene duplication since the split from the rodent lineage. Strikingly, nearly all of the CYP450 enzymes with known xenobiotic substrates were found in these gene clusters (e.g., see Figure 1 chromosome positions). The remaining genes in these clusters were also phylogenetically unstable but are not yet implicated in xenobiotic detoxification. Each of the human clusters had a syntenic counterpart cluster in mouse (Figures S1, S3, and S4). Very few of the genes in these clusters could be assigned as one-to-one orthologs because of repeated duplication and deletion events on both lineages. These syntenic gene clusters must have originated from a shared ancestral gene or genes, with repeated local gene duplications and losses giving rise to lineage-specific groups of related genes. The patterns of divergence are consistent with single gene duplications that occurred at various times, though multigene duplications followed by loss of most of the duplicate genes cannot be ruled out. Genomic clustering of related genes was also apparent in the other eight genomes, but assembly quality was more problematic, and I did not investigate the patterns in detail. These results are consistent with increased rates of birth-death evolution specific to xenobiotic-substrate genes, with local gene duplications giving rise to clusters of related genes.

### Many Unstable Genes Are Subject to Positive Selection

If exposure to xenobiotic compounds changed over evolutionary time, the *CYP450* detoxifier genes might be subject to positive selection driven by changing substrates. To test this possibility, I used maximum-likelihood codon analysis on paralogous groups of genes from many parts of the overall CYP450 tree [14,15]. Most attention was given to tree regions where there was evidence of frequent recent gene duplication, both because this might indicate selection to diversify enzyme function and because this provided abundant sequence data for analysis. Specifically, 203 genes in clades with five or more closely-related genes were tested, including six groups from primates, nine from rodents, and six from a mixture of primates and rodents. Details of the method are given in Materials and Methods, and summary statistics for the results are given in Table S3. A total of five of the 21 tested sets showed highly significant evidence of positive selection. The specific amino acid sites under positive selection were analyzed for a set of 12 rodent *Cyp2d* genes, which showed the strongest signal of positive selection. In this set, a Bayes-Empirical-Bayes method identified nine sites with strong evidence of positive selection (*p* > 0.9 for each site). The structural positions of these nine sites were compared by alignment with two closely related CYP2 proteins for which crystal structures have been determined [16,17]. Both in the primary sequence alignment and in the 3-D protein structure, the nine sites under positive selection were clearly associated with substrate-binding or substrate-channel regions (Figures S6 and S7). In the protein structure, the distance from positive selection sites to a bound warfarin ligand was significantly smaller than for random sets of sites (*p* ≈ 0.0002, see Materials and Methods). In addition to these nine sites, all three small indels among the 12 rodent genes coincided with regions of substrate binding (Figure S6). No method exists to analyze such indels for positive selection, but it is plausible that they affect substrate interactions and might be subject to such selection. These results are consistent with changing xenobiotic substrates driving the observed positive selection.

For comparison with the unstable genes, nine sets of genes from stable *CYP450* groups were also tested for positive selection (marked on Figure S1). In each case, the orthologous genes from the six mammals were tested as a group. Because there are almost no gene duplications in these groups, it was not possible to include paralogs in these sets, but the degree of divergence of the genes (total tree length) was in the same range as for the paralog groups, indicating that the maximum-likelihood analysis should have similar power [18]. No evidence of positive selection was found for any of the nine sets, as expected based on their function as enzymes acting on endogenous substrates.

## Discussion

Phylogenetic instability among genes in families has been observed repeatedly, including among immunoglobulin genes [19], major histocompatibility complex genes [20], T-cell receptor genes [20,21], mammalian olfactory receptor genes [22], nematode chemosensory genes [15,23], globin genes [24,25], cichlid opsin genes [26,27], zinc-finger genes [28], F-box and MATH-BTB genes [29], and others. Selective pressure from environmental change or pathogens has been suggested as a driver of duplication and diversification for many of these examples, but alternatives are difficult to rule out. The vertebrate *CYP450* gene superfamily affords special insight into gene instability because probable biochemical functions of most of the genes are known and because it includes both stable and unstable genes. I found a strong correspondence between genes implicated in xenobiotic detoxification and evolutionary instability in the form of gene duplication and deletion. These findings strongly support the involvement of changing external selective pressure in high rates of birth-death gene evolution. Positive selection for amino acid change has been also been reported in many of the phylogenetically unstable gene families, suggesting that diversification is achieved by a combination of gene duplication and selection-driven divergence in sequence.

For other unstable gene families, lack of stable family members for comparison or lack of extensive functional information prevent the highly informed analysis possible for the *CYP450* genes. Nevertheless, a similar explanation for birth-death evolution is plausible for most or all such families. Immunoglobulin genes, major histocompatibility complex class I and class II genes, and T cell receptor genes function in pathogen defense, and an extensive literature documents gene duplications and deletions in these families during vertebrate evolution. Mammalian olfactory receptor genes and nematode chemosensory genes function in response to environmental chemical stimuli and might be subject to changes in desired ligand specificity or sensitivity. Lake Malawi cichlid fish have undergone duplications of opsin genes that have diverged to different spectral sensitivities, and expression of specific sets of these genes results in diverse adaptive visual properties, possibly driven by mate choice and foraging preferences [26,27]. In other cases the role of external selective forces in driving birth-death evolution is less obvious but plausible; for example the F-box and MATH-BTB genes in nematodes and plants have been hypothesized to function in pathogen defense, though the evidence is indirect [29].

Much less is known about specific functions of *CYP450* genes in nematodes and plants. Based on maximum-likelihood protein trees with three *Caenorhabditis* species *(briggsae, elegans,* and *remanei)* and with the plants Arabidopsis thaliana and *Oryza sativa* (rice), it is clear that birth-death evolution affects most members of the CYP450 family in these groups as well. For example, only 7% of *Arabidopsis CYP450* genes and 38% of C. elegans genes had strict one-to-one orthologs within their groups (unpublished data). As in the human and mouse genomes, closely related unstable genes tended to be in genomic clusters. The exact degrees of stability and instability cannot be directly compared because the phylogenetic distances differ, and genome annotation qualities may vary.

A few *CYP450* genes in humans lack clear functional information. The relative evolutionary stabilities of these genes may be predictive of general aspects of their functions. Among stable genes of unknown function, human *CYP20A1* and orthologs are well separated from all other *CYP450* genes on the protein tree and are encoded by a single gene in each of the ten species analyzed (top left of Figure S1). This pattern suggests an ancient and critical function that is shared among all the species. A less clear-cut but similar pattern is seen for an unnamed human *CYP27* (Chromosome 2) and the *CYP2U1* and *CYP2R1* genes. I speculate that these four enzymes will prove to be involved in biosynthesis or catabolism of endogenous substrates of importance throughout vertebrates. Consistent with this interpretation, *CYP20A1* expression is enriched in immune system cell types, *CYP2U1* is expressed broadly, and *CYP2R1* expression is enriched in testis and immune system [30]. Unstable genes for which I could find no described function include *CYP4A43, CYP4Z1, CYP4Z2* (possible pseudogene), *CYP4X1, CYP4A11, CYP4A22, CYP4F2, CYP4F3, CYP4F8, CYP4F11,* and *CYP4F12*. Among these, in addition to birth-death evolution, the *CYP4F* group is subject to significant positive selection, consistent with substrate-driven selection for amino acid change. I speculate that each of these genes functions in xenobiotic detoxification or some other process that acts on environmental substrates. Consistent with this interpretation, expression of most of these genes is enriched in liver *(CYP4A11, CYP4A22, CYP4F2, CYP4F3, CYP4F11,* and *CYP4F12)* or in tracheal tissue *(CYP4X1)* [30], which are tissues where other xenobiotic *CYP450* genes are known to function [6,7]. Finally, the evolution of the *CYP2W1* and *CYP2J2* genes is less clear, with duplications in some species but only a single gene in humans. *CYP2W1* has a broad expression pattern [30], and evolution of the group appears most consistent with a relatively stable function in mammals but extensive duplication and diversification in amphibians and teleost fish. *CYP2J2* expression is enriched in liver [30], and the group includes multiple genes in most species but only a single gene in humans and macaques.

Groups of closely related unstable *CYP450* genes are strongly clustered in the human and mouse genomes. A similar pattern of genome clustering is found in a number of other human and mouse gene families [31–33]. These patterns indicate that DNA duplications that persist in the genome occur predominantly locally. In contrast, about half of very recent large DNA duplications (segmental duplications) on the human lineage are interchromosomal, and some of the remainder occur nonlocally on the same chromosome [34–37]. It is possible that these segmental duplications represent a gene duplication mechanism that has dramatically and recently increased on the primate lineage [34]. Alternatively, nonlocal segmental duplications may be more unstable than local duplications and fail to persist over longer evolutionary periods, so that they rarely give rise to persistent functional duplicate genes.

The combination of evolutionary instability and gene clustering has parallels with “genomic islands” found in bacteria. Genomic islands consist of blocks of DNA that are present in some bacterial isolates but absent from other closely related isolates (reviewed in [38]). Though the full picture is still developing, it is likely that most genes in genomic islands have auxiliary organismal functions such as pathogenesis, antibiotic resistance, symbiosis, and specialized metabolism [38]. Despite some parallels, the mechanisms by which bacterial genomic islands and unstable gene islands in animals arise are different: clustering in bacteria is a consequence of horizontal transfer of contiguous blocks of DNA, whereas clustering in animals is restricted to closely-related genes and arises from local gene duplication events without horizontal gene transfer.

## Materials and Methods

### Sequence datasets.

Sequence data were downloaded from ENSEMBL [39] release 40, which contained the following genome builds: H. sapiens NCBI36, M. mulatta MMUL1, B. taurus Btau2.0, C. familiaris BROADD1, M. musculus NCBI36, R. norvegicus RGSC3.4, G. gallus WASHUC1, X. tropicalis JGI4.1, D. rerio ZFISH6, and T. rubripes FUGU4.40. Data for P. troglodytes were not included because the genes are too similar to human to be informative. Data for Monodelphis domestica and Tetraodon nigroviridis and several low coverage genomes were considered but rejected because current gene prediction sets appeared to be too incomplete or inaccurate (unpublished data). Starting with a hand-collected set of human CYP450 sequences, all known isoforms of human CYP450 proteins were collected from a protein BLAST (BLASTP) search of all human proteins. Many genes had multiple transcript and protein forms. Each such case was analyzed to identify a single protein isoform that appeared full length and most family-typical, based on alignment with related CYP450 proteins. This analysis resulted in a set of 60 reference human proteins that were full length or nearly full length, including three possible pseudogenes. Proteins from other species were collected from BLASTP searches with these 60 human proteins as query. All isoforms were kept initially, and for each gene the isoform with the highest BLASTP score against a human protein was kept for further analysis (for the most part, this approach discarded obviously incomplete proteins). Subsequent tree analysis of these proteins indicated the surprising absence of a few CYP450 proteins with endogenous substrates. Some of these were found to be present (but unpredicted) in the cognate genomes by TBLASTN searches (Table S1). Other genes are probably present in unassembled sequence; the number of missing genes and genome sequence coverage in the mammalian genomes is summarized in Table S2.

### Protein trees.

After discarding a few proteins that were missing more than 30% of the expected CYP450 protein or clearly aligned badly, the remaining set of 628 proteins were aligned with ClustalW (default settings except gap clustering turned off) [40]. A complete list of these protein sequences is found in Dataset S1 with ENSEMBL gene identifiers [39]. After removal of aligned columns with more than 30% gap residues, the resulting multiple alignment had 417 sites, at least 70% of which were present in any given sequence. A maximum-likelihood tree was constructed from this alignment with PhyML (JTT matrix, four rate categories, gamma shape parameter 1.0) [41]. Tree nodes were rotated, colored, and displayed with *Bonsai* [42]. Additional annotations were added to the tree image for Figure S1. The complete tree was subjected to 200 bootstrap repeats with PhyML with the same settings, which required ten days of central processing unit time. Subtrees of specific interest were tested with 1,000 bootstrap repeats (Figure S2).

### Codon analysis for positive selection.

Multiple sets of five to 15 closely related primate and rodent genes were selected from the tree in Figure S1. For each set, a protein alignment was made using ClustalW (default settings except gap clustering turned off) [40]. This protein alignment was used to generate the corresponding codon alignment and to construct a maximum-likelihood protein tree with PhyML [41]. The tree and codon alignment were analyzed with CodeML from PAML 3.14 [14], using models 7 and 8, with three starting *d*_N_/*d*_S_ (ω) values for model 8 to avoid local optima. The neutral model 7 assumes a β-distribution of 10 *d*_N_/*d*_S_ ratio classes constrained to lie between 0 and 1.0, whereas the selection model 8 permits one additional *d*_N_/*d*_S_ ratio class without constraint. In order to minimize the effects of gene prediction and alignment errors, aligned columns with a gap in any sequence were excluded from analysis (“cleandata” option in CodeML). The transition/transversion ratio (κ) was estimated by CodeML. Statistical significance was assessed using a χ-square test on twice the difference in log-likelihood values (ΔML) for models 7 and 8 with two degrees of freedom, a statistic shown to be conservative in simulations [43]. Assignment of specific sites under likely positive selection was based on the Bayes-Empirical-Bayes test implemented in CodeML. In Figures S6 and S7, marked sites are subject to positive selection with *p* > 0.9. Extensive gene conversion can exaggerate signals of positive selection, but direct analysis of gene conversions among the tested genes suggests that they are unlikely to confound the analysis. GENECONV [44] was used to test for possible gene conversion events in the complete genomic regions of all the mouse and human genes analyzed for positive selection (unpublished data). In all cases except one, detected regions of conversion affected only a few genes in <10% of their coding region, suggesting that they will not significantly affect the signal of positive selection. The exception was among mouse genes in the *CYP2D* family, but even in this case a minority of genes had evidence of gene conversion and none were affected in more than 20% of their coding region.

### Structure analysis.

The significance of the association of positive selection sites to bound warfarin ligand in the *CYP2C9* structure was assessed as follows. The mean physical distance of the alpha backbone carbon atom to a reference warfarin atom was measured for the nine amino acid residues under positive selection. Warfarin atom C10 (carbon 10, atom 7439 of Protein Data Base structure 10G5) was used as the reference; it was selected as centrally located in the binding pocket/access channel based on visual inspection of the three-dimensional protein structure. The expected mean distance for randomly located amino acid residues was determined by repeatedly choosing nine random amino acid residues and computing the mean distance from their alpha backbone carbon atoms to the reference warfarin atom. Mean distances for 10,000 simulation repeats fitted a normal distribution (Figure S8), and all but two had mean distances larger than the mean distance for positively selected sites.

## Supporting Information

Dataset S1 Protein Sequences (315 KB XLS)

Figure S1 Maximum-Likelihood Tree of 628 Vertebrate CYP450 Proteins Filled black square: human protein with known endogenous substrate. Filled blue circle: human protein with known xenobiotic substrates. Red asterisks indicate positive selection was detected; tests for positive selection were performed only on primate and rodent genes. Other markings indicate enzyme activity, gene clusters in human (blue) and mouse (pink), family groups (black), and specific groups tested where positive selection was not found (green). References for specific endogenous-substrate gene functions are as follows. CYP26C1 (retinoic acid catabolizing enzyme) [45]; CYP26B1 (retinoic acid catabolizing enzyme) and CYP26A1 (retinoic acid 4-hydroxylase) [46]; CYP51A1 (sterol 14-α-demethylase) [47]; CYP39A1 (oxysterol 7-α-hydroxylase) [48]; CYP8A1 (prostacyclin synthase) [49]; CYP8B1 (sterol 12-α-hydroxylase) [50]; CYP7B1 (oxysterol 7-α-hydroxylase) [51,52]; CYP7A1 (cholesterol 7-α-hydroxylase) [53,54]; CYP19A1 (estrogen synthetase or aromatase) [55]; CYP46A1 (cholesterol 24-hydroxylase) [56]; CYP5A1 (thromboxane-A synthase) [57,58]; CYP4V2 (fatty-acid metabolism) [59]; CYP24A1 (vitamin D3 24-hydroxylase) [60]; CYP11A1 (cholesterol side-chain cleavage enzyme) [61]; CYP11B2 (aldosterone synthase) and CYP11B1 (steroid 11-β-hydroxylase) [62]; CYP27A1 (sterol 26-hydroxylase) [63]; CYP27B1 (25-hydroxyvitamin D3 1 α-hydroxylase) [64]; CYP21A2 (steroid 21-hydroxylase) [65]; CYP17A1 (steroid 17-α-monooxygenase) [66]; CYP1B1 (steroid catabolism) [67]. Evidence for xenobiotic activity of CYP450 enzymes is often scattered among many papers; the markings here are based primarily on two recent reviews [6,7].
(2.6 MB JPG)

Figure S2 Bootstrap Tests on Subtrees A total of four sets of proteins that form clades on the tree in Figure S1 were tested by more extensive bootstrap analysis. The four sets are from the CYP3A, CYP4F, CYP2C, and CYP2A/B families. In each case, the proteins from the family were aligned and subjected to 1,000 bootstrap repeats as described in Materials and Methods. Bootstrap values shown (percentage branch support) are those that are relevant to confidence of species-specific gene duplications. In a separate analysis, two outgroup proteins were added to the set for rooting.
(1.0 MB JPG)

Figure S3 Relationship of Genes Within and among the Mouse and Human CYP2C Unstable Gene Islands The figure is derived from snapshots on the University of California Santa Cruz Genome Browser (http://genome.ucsc.edu) in October 2006. Orthologous gene pairs flanking the unstable *CYP450* genes are marked with green lines, which include one pair of *CYP450* genes (human *CYP2C18* and mouse *Cyp2c55* at the left end of the unstable island). ENSEMBL gene identifiers are shown for two genes without formal mouse gene names. The unstable genes in the island include three human *CYP450* genes and 12 mouse *Cyp450* genes. A protein tree relating all the genes in these clusters is shown in Figure S2 (bottom left).
(599 KB JPG)

Figure S4 Relationship of Genes within and among the Mouse and Human CYP3A Unstable Gene Islands See Figure S3 legend description. This gene cluster underwent a single rearrangement on the mouse lineage that split the mouse cluster into two parts, which are shown separately aligned to the human cluster. The unstable genes in the island include four human *CYP450* genes and nine mouse *Cyp450* genes. Positions of two ENSEMBL predicted mouse *Cyp450* genes that did not appear on the UCSC browser were added by hand.
(575 KB JPG)

Figure S5 Orthology and Synteny among Human and Macaque CYP3A Genes Human genes are labeled in blue and macaque genes are labeled in green. Both species have four *CYP3A* genes that are present in a single gene cluster, and gene pairs marked syntenic occupy equivalent positions in their respective clusters. The tree is inconsistent with significant concerted evolution subsequent to separation of the two lineages. The proteins encoded by each gene were aligned and subjected to maximum-likelihood tree analysis with 1,000 bootstrap repeats as described in Materials and Methods. Bootstrap values shown (percentage of branch support) are those relevant to determining orthology. A total of two of the macaque genes were not predicted in the current assembly (MMUL1); they were identified by TBLASTN searches with the human protein as query. For each, a gene prediction was hand assembled, and the protein is labeled with chromosome and position information in place of a gene name.
(260 KB JPG)

Figure S6 Positive Selection Sites Mapped onto a Rodent CYP2D Alignment Full length alignments of proteins from the 12 rodent genes with the strongest signal of positive selection and two closely related human proteins with crystal structures. Blue shading is proportional to sum-of-pairs conservation for each residue with respect to its aligned column. Major alpha helices are marked above the alignment for context. Sites with significant evidence of positive selection (*p* > 0.9) are marked with red asterisks and numbered below the alignment; numbers correspond with those in Figure S7. A total of three sites of indels among the rodent genes are marked with a red D. Residues that line the substrate-binding cavity in the crystal structures are marked with an orange S, and residues that form the substrate-entry channel are marked with an orange C. Heme-binding residues are marked with a green H. The binding site, entry channel, and heme-binding assignments are based solely on the published description [17]. Positive selection site 1 is also located close to the substrate-entry channel (Figure S7).
(2.3 MB JPG)

Figure S7 Positive Selection Sites Mapped onto the CYP2C9 Structure Red space-filled residues align with sites of positive selection in the set of proteins in Figure S6. The alignment positions of two indel sites are marked on the backbone with red arrowheads; the third indel site is hidden behind the residue marked 9 on a loop that forms part of the substrate-binding cavity. The bound warfarin ligand is shown in purple, and the heme is shown in brown. A second probable substrate-binding site is located between the warfarin and the heme but is unoccupied in this structure [17]. Alpha helices are shown by gray cylinders, and their lettering corresponds to that in Figure S6. The olive section of backbone is membrane associated.
(600 KB JPG)

Figure S8 Distribution of Mean Distances from Random Sampling of CYP2C9 The mean distance to the reference warfarin atom C10 from 10,000 samples of nine random residues from the CYP2C9 structure is shown. The sampled distances were put into bins of 1 Angstrom for graphing. The black arrow indicates the mean distance for the nine sites of positive selection to the same warfarin atom (14.4 Angstroms).
(164 KB JPG)

Table S1 Results of TBLASTN Fishing for Unpredicted Genes For each case of an expected mammalian gene that was not in the predicted set for a genome, the protein from another species was used as query for a TBLASTN search of the genome (−m 9 option, no filtering, E-value cutoff 10). For cases where no good match was found, the table has an entry giving only the query and the result (in most cases there were no matches with an E-value < 10^−3^). For cases where a strong candidate gene was found, the TBLASTN output is shown, with unrelated matches removed and sorted by query position.
(16 KB XLS)

Table S2 Summary of Probable Missing Stable Genes Summary of gene content among mammals for the 20 most stable groups of CYP450 enzymes with known endogenous substrates (all proteins marked with a black square on Figure S1, except for the pair of corticosteroid enzymes CYP11B1 and CYP11B2). A total of 20 genes are expected for each genome, but one to three genes were missing from predicted gene sets for all the genomes except human and mouse. Column 3 summarizes the results from Table S1 (putative genes found by TBLASTN searches), and column 4 indicates the number of genes that remain missing. Column 5 summarizes the estimated single-copy sequence coverage for each genome, based on NCBI genome summaries. Not all of this sequence is present in the assemblies used for TBLASTN searching.
(14 KB XLS)

Table S3 Summary of Statistics for Maximum-Likelihood Analysis of Positive Selection In each column the CYP family, coding sequence file name, number, and mean length of sequences are given, followed by analysis results relevant to inferring positive selection. For cases in which the difference in maximum-likelihood for models 7 and 8 was large, a *p*-value was computed (see Materials and Methods) with Bonferroni correction. Total tree length is a significant parameter for sensitivity [43]. The “model 8 11th class omega” value is the value for the free *d*_N_/*d*_S_ class in model 8, which was much greater than 1.0 in all cases of high significance.
(18 KB XLS)

### Accession Numbers

The Protein Data Base (http://www.pdb.org) number for the structure discussed in this paper is 1OG5.

## Abbreviations

*CYP450*: cytochrome P450
TBLASTN: translated BLAST nucleotide
